# Prevalence of HIV self-testing and associated factors among men who have sex with men in Chongqing, China: a cross-sectional study

**DOI:** 10.3389/fpubh.2026.1774567

**Published:** 2026-04-08

**Authors:** Tianyu Tan, Bo Tan, Guohui Wu, Chao Zhou, Long Li, Chongyang Bai, Jing Lin, Yaping He, Hehong Jiang, Wei Zhang, Rongrong Lu, Xingjian Luo

**Affiliations:** Chongqing Center for Disease Control and Prevention (Chongqing Academy of Preventive Medicine), Chongqing, China

**Keywords:** associated factors, China, HIV, HIV self-testing, men who have sex with men

## Abstract

**Background:**

HIV self-testing has been promoted globally as an effective complement to facility-based testing, particularly among men who have sex with men (MSM). However, evidence on HIV self-testing uptake and its determinants in western China remains limited.

**Objective:**

To assess the prevalence of HIV self-testing and identify associated factors among MSM in Chongqing, China.

**Methods:**

A cross-sectional study was conducted from March 2021 to March 2022 among MSM recruited using a snowball sampling strategy at CDC-based voluntary counseling and testing clinics in Chongqing. Participants completed an anonymous face-to-face questionnaire covering socio-demographic characteristics, HIV-related knowledge, sexual behaviors in the past 6 months, substance use, and HIV self-testing history. Multivariate logistic regression was used to identify factors associated with HIV self-testing.

**Results:**

A total of 2,315 MSM were included. Overall, 16.2% (374/2,315) reported ever using HIV self-testing kits, with blood-based tests accounting for 97.6% of use. Higher educational attainment (high school/vocational school: *OR* = 2.56; college or above: *OR* = 3.60), having two or more sexual partners in the past 6 months (*OR* = 11.85), and use of sexual stimulants (*OR* = 3.49) were positively associated with HIV self-testing. Being married or cohabiting was negatively associated with HIV self-testing (*OR* = 0.30).

**Conclusion:**

HIV self-testing uptake among MSM in Chongqing remains low. Education level, marital status, sexual behavior, and stimulant use were key correlates. These findings underscore the need to integrate HIV self-testing into existing HIV prevention services, with targeted strategies for MSM with lower educational attainment and those in stable partnerships.

## Introduction

HIV infection continues to pose a major global public health challenge. According to the Joint United Nations Programme on HIV/AIDS, approximately 40.8 million people were living with HIV worldwide in 2024, with nearly 87% aware of their infection status (1). Despite substantial progress, gaps in HIV diagnosis persist in many countries. In China, an estimated 20% of people living with HIV remain unaware of their infection, indicating that the first target of the UNAIDS “95–95–95” goals has not yet been achieved (2, 3).

Men who have sex with men (MSM) are one of the populations most affected by HIV (4, 5). In China, the proportion of newly reported HIV infections attributed to MSM has increased markedly over the past two decades (6), highlighting the urgent need for effective testing and prevention strategies tailored to this group. Early diagnosis through regular HIV testing remains the cornerstone of HIV prevention and treatment, as it enables timely linkage to care and reduces onward transmission.

Although China has implemented extensive facility-based HIV testing services, including free voluntary counseling and testing (7), uptake among MSM remains suboptimal. Barriers such as stigma, concerns about confidentiality, inconvenient service hours, and discomfort with clinical settings continue to limit testing coverage (8, 9). HIV self-testing, which allows individuals to collect samples, perform tests, and interpret results privately, offers a promising alternative, has been widely promoted and extensively utilized among MSM populations (10, 11). HIV self-testing has been shown to increase testing frequency, reach first-time testers, and reduce testing-related barriers among MSM (12, 13).

While HIV self-testing has been increasingly promoted in China, most existing studies have focused on coastal or economically developed regions (14–16). Evidence from western China, particularly large municipalities such as Chongqing, remains limited. Compared with coastal metropolitan areas in China, western regions are characterized by relatively uneven economic development, large internal migration flows, and disparities in access to community-based sexual health resources (17). In addition, expansion of HIV self-testing distribution channels in recent years and the reorganization of health services following the COVID-19 pandemic may have reshaped testing pathways for MSM (18). Updated data from Chongqing are therefore essential to understand how these contextual factors influence HIV self-testing uptake in an under-documented but epidemiologically important setting. Therefore, this study aimed to assess the uptake of HIV self-testing and identify associated factors among MSM in Chongqing, China.

## Objects and methods

### Study design and participants

This cross-sectional study was conducted from March 2021 to March 2022 in Chongqing, China. Participants were recruited at AIDS counseling clinics of the Chongqing Municipal Center for Disease Control and Prevention and the Jiangbei District Center for Disease Control and Prevention.

Eligibility criteria included: (1) age ≥ 18 years; (2) history of anal or oral sex with another man in the past year; (3) ability to provide informed consent and complete the questionnaire independently. Individuals with severe mental disorders or inability to complete the questionnaire were excluded. The age criterion of 18 years or older was applied in accordance with national ethical regulations regarding the legal capacity to provide informed consent for HIV-related surveys.

### Sampling and data collection

Snowball sampling was adopted because MSM represent a hidden and stigmatized population in China, making probability-based sampling difficult to implement. This strategy has been widely used in HIV research to reach individuals who may otherwise remain inaccessible through routine recruitment approaches (4). After providing informed consent, participants completed a face-to-face anonymous questionnaire administered by trained staff in a private setting. The questionnaire collected information on socio-demographic characteristics, HIV/AIDS knowledge, sexual behaviors in the past 6 months, substance use (including sexual stimulants), and history of HIV self-testing. HIV/AIDS-related knowledge was assessed using eight standardized questions from the National HIV Sentinel Surveillance Questionnaire, with six or more correct answers indicating adequate knowledge.

Following the survey, blood samples were collected for HIV and syphilis antibody testing according to standard laboratory procedures.

### Measures

HIV self-testing was defined as ever having used an HIV self-testing kit, regardless of testing frequency or timing. Sexual stimulant use referred to the use of aphrodisiacs or recreational substances to enhance sexual experience in the past 6 months. Information was collected as a composite measure, and specific types of substances were not differentiated.

### Quality control

All investigators received standardized training prior to data collection. Logical checks were embedded in the questionnaire system to reduce entry errors. Completed questionnaires were reviewed on-site for completeness and consistency, and any discrepancies were clarified immediately.

### Statistical analysis

Data were analyzed using SPSS version 25.0. Descriptive statistics were used to summarize participant characteristics. Chi-square tests were conducted to examine differences in HIV self-testing across subgroups. Variables with *p* < 0.05 in univariate analyses were entered into a multivariate logistic regression model to identify factors independently associated with HIV self-testing. Odds ratios (ORs) and 95% confidence intervals (CIs) were reported, with *p* < 0.05 considered statistically significant. However, selecting variables based on statistical significance in univariate analyses is a commonly used approach; however, this strategy may overlook potential conceptual confounders. Therefore, findings should be interpreted with caution.

## Results

### Participant characteristics

A total of 2,315 MSM were included in the analysis. The mean age of participants was 32.6 years (standard deviation: 10.8), and 46.2% were aged 25–34 years. Most participants were unmarried (72.2%) and identified as homosexual (80.0%). Approximately 64.8% had a college degree or higher, and 87.9% demonstrated adequate HIV/AIDS-related knowledge (Table 1).

**Table 1:** Characteristics, sexual behaviors and substance use of MSM in Chongqing.

Variables	Number	Proportion (%)
Age group		
≤ 24	526	22.7
25~34	1,069	46.2
≥35	720	31.1
Marital status		
Unmarried	1,672	72.2
Marriage/Cohabitation	459	19.8
Divorced or widowed	184	7.9
Degree of education		
Junior high school and below	375	16.2
High school or vocational school	441	19.0
College degree or above	1,499	64.8
Character		
Insert a square	520	22.5
Inserted party	941	40.6
Both	854	36.9
Sex orientation		
Homosexuality	1,852	80.0
Bisexuality	463	20.0
Age group at first male–male sex		
≤ 17	277	12.0
18~24	1,456	62.9
≥25	582	25.1
Number of sexual partners in the past 6 months		
1 or fewer	2,278	98.4
2 or more	37	1.6
Drug use		
Yes	12	0.5
No	2,303	99.5
Use of sexual stimulants		
No	2,030	87.7
Yes	285	12.3
HIV/AIDS-related knowledge		
No	280	12.1
Yes	2,035	87.9
HIV antibody positive		
Yes	291	12.6
No	2,024	87.4
Syphilis antibody positive		
Yes	412	17.8
No	1,903	82.2

### Sexual behaviors and substance use

The mean age at first male–male sexual intercourse was 22.4 years (standard deviation: 6.8). In the past 6 months, 1.6% of participants reported having two or more sexual partners. Use of sexual stimulants during the same period was reported by 12.3% of participants (Table 1).

### HIV self-testing uptake

Overall, 16.2% (374/2,315) of participants reported ever using HIV self-testing kits. Among them, blood-based self-testing kits were the most commonly used (97.6%), while saliva-based and urine-based kits were used less frequently (Table 2).

**Table 2:** Univariate analysis of HIV self-testing among MSM in Chongqing.

Variables	HIV self-testing (%)	Single factor analysis	
		χ^2^	*P*
Age group			
≤ 24	101 (19.2)	12.137	0.002
25~34	184 (17.2)		
≥35	89 (12.4)		
Marital status			
Unmarried	327 (19.6)	55.541	< 0.001
Marriage/cohabitation	25 (5.4)		
Divorced or widowed	22 (12.0)		
Degree of education			
Junior high school and below	16 (4.3)	57.546	< 0.001
High school or vocational school	59 (13.4)		
College degree or above	299 (19.9)		
Character			
Insert a square	99 (19.0)	6.555	0.038
Inserted party	132 (14.0)		
Both	143 (16.7)		
Sex orientation			
Homosexuality	322 (17.4)	10.361	0.001
Bisexuality	52 (11.2)		
Age group at first male–male sex			
≤ 17	51 (18.4)	3.044	0.218
18~24	241 (16.6)		
≥25	82 (14.1)		
Number of sexual partners in the past 6 months			
1 or fewer	348 (15.3)	81.290	< 0.001
2 or more	26 (70.3)		
Drug use			
Yes	0		0.234
No	374 (16.2)		
Use of sexual stimulants			
No	259 (12.8)	140.465	< 0.001
Yes	115 (40.4)		
HIV/AIDS-related knowledge			
No	27 (9.6)	9.974	0.002
Yes	347 (17.1)		
HIV antibody positive			
Yes	49 (16.8)	0.115	0.735
No	325 (16.1)		
Syphilis antibody positive			
Yes	78 (18.9)	2.852	0.091
No	296 (15.6)		

### Factors associated with HIV self-testing

In multivariate logistic regression analysis, higher educational attainment was significantly associated with HIV self-testing, with increased odds observed among participants with high school/vocational education and those with college education or above. Having two or more sexual partners in the past 6 months and use of sexual stimulants were also positively associated with HIV self-testing. In contrast, being married or cohabiting was negatively associated with HIV self-testing. HIV/AIDS-related knowledge was not independently associated with HIV self-testing after adjustment for other variables (Table 3).

**Table 3:** Multivariate logistic regression analysis of MSM HIV self-testing in Chongqing.

Variables	Multiplicity		
	OR	95% CI	*P*
Age group			
≤ 24	1	–	–
25~34	0.862	0.645~1.152	0.315
≥35	1.096	0.749~1.604	0.638
Marital status			
Unmarried	1	–	–
Marriage/cohabitation	0.297	0.184~0.478	< 0.001
Divorced or widowed	0.700	0.402~1.220	0.209
Degree of education			
Junior high school and below	1	–	–
High school or vocational school	2.561	1.413~4.644	0.002
College degree or above	3.600	2.087~6.210	< 0.001
Character			
Insert a square	1	–	–
Inserted party	0.877	0.642~1.198	0.409
Both	0.858	0.630~1.168	0.330
Sex orientation			
Homosexuality	1	–	–
Bisexuality	0.863	0.614~1.211	0.393
Number of sexual partners in the past 6 months			
1 or fewer	1	–	–
2 or more	11.848	5.311~26.430	< 0.001
Use of sexual stimulants			
No	1	–	–
Yes	3.490	2.618~4.653	< 0.001
HIV/AIDS-related knowledge			
No	1	–	–
Yes	1.452	0.928~2.272	0.102

## Discussion

From a behavioral perspective, uptake of HIV self-testing may reflect perceived susceptibility, perceived benefits, and barriers to service access, as described in health behavior theories such as the Health Belief Model (19). In western China, structural conditions including economic diversity, migration, and unequal distribution of community resources may further shape how MSM evaluate different testing options (17). Moreover, temporary restrictions on facility-based services during local COVID-19 outbreaks might have increased the attractiveness of private and flexible testing approaches such as self-testing (18).

This study assessed the prevalence of HIV self-testing and its associated factors among MSM in Chongqing, China. The findings indicate that HIV self-testing uptake remains relatively low, despite slightly higher than the 2017 survey result (20). Compared with studies conducted in eastern and coastal regions of China (21–23), the prevalence observed in Chongqing was substantially lower, suggesting regional disparities in HIV self-testing accessibility and acceptance.

We found that marital/cohabitation status was negatively associated with HIV self-testing, aligns with previous research (24, 25). MSM in stable relationships may avoid testing due to concerns about partner discovery, potential relationship disruption, and reduced risk awareness. Despite this, these individuals remain at high risk of HIV infection (26). This finding highlights the need for discreet and stigma-free testing options tailored to MSM in stable relationships.

Higher educational attainment was positively associated with HIV self-testing, consistent with findings from other studies (27–29). Individuals with higher education may have better access to health information, greater awareness of HIV prevention strategies, and higher confidence in using self-testing kits (30, 31). Targeted interventions are therefore needed to improve HIV self-testing uptake among MSM with lower educational levels.

Multiple sexual partners and sexual stimulant use are linked to high-risk sexual behaviors (32–34). The strong association between having multiple sexual partners, sexual stimulant use, and HIV self-testing suggests that individuals engaging in higher-risk behaviors may have greater risk perception and motivation to test, aligns with most research (35, 36). Integrating HIV self-testing with behavioral risk reduction interventions may further enhance its public health impact. However, only a small proportion of participants reported having two or more sexual partners. Sparse data in this category may have produced unstable estimates and inflated odds ratios. Therefore, the magnitude of this association should be interpreted cautiously.

The loss of statistical significance after multivariate adjustment may suggest that the effect of HIV-related knowledge is mediated by factors such as education level or prior engagement with health services (37). In other words, knowledge alone may be insufficient to translate into testing behavior without supportive structural conditions (38).

The high usage rate of blood-based self-testing kits (97.6%) compared to saliva (14.2%) and urine (1.3%) kits aligns with findings by Shan et al. (39). This may relate to price differences, as blood-based kits are generally more affordable. Cost is a significant barrier to obtaining self-testing kits (40, 41), and providing free kits can substantially increase testing frequency (42). It is important to emphasize that HIV self-testing serves as a supplement to traditional testing rather than a replacement, as results provide preliminary information and should not be used as a final diagnosis (43).

More locally tailored implementation strategies may be required in Chongqing. Discreet mail-delivery of self-testing kits could improve accessibility for married MSM or those concerned about unintended disclosure (44). For individuals with lower educational attainment, pictorial or video-based instructions and community-assisted demonstrations may enhance correct usage and confidence (45). Strengthening collaboration between CDC services and community-based organizations may also facilitate distribution, follow-up, and referral (46).

### Limitations

Several limitations should be noted. First, the cross-sectional design precludes causal inference. Second, because recruitment partly relied on VCT clinics and peer referral, MSM who were already connected to health services or possessed higher health literacy may have been over-represented. Studies comparing clinic-based and online samples in China suggest that facility-recruited participants often report greater testing experience (47). Consequently, our findings may not fully reflect more hidden subgroups. Third, behavioral data were self-reported and may be subject to recall and social desirability biases. In addition, sexual stimulant use was measured as a combined category, and we were unable to distinguish between different substances. Because various drugs may influence risk perception and testing motivation in different ways, more detailed classification should be incorporated in future research. Furthermore, we did not collect information on linkage to confirmatory testing or treatment among individuals with reactive results. As a result, the contribution of HIV self-testing to subsequent stages of the care cascade could not be evaluated.

### Strengths

Despite these limitations, this study provides updated evidence from a large sample of MSM in a major municipality of western China. Unlike earlier studies conducted in Chongqing and other regions of China, this study was carried out after the broader promotion of HIV self-testing policies and during a period marked by changes in healthcare access following the COVID-19 pandemic. By using a large clinic-based sample and examining both behavioral and contextual factors, this study provides updated evidence on HIV self-testing uptake among MSM in a major municipality in western China, offering insights into persistent gaps and priority subgroups for intervention. Although linkage to confirmatory diagnosis and treatment is critical for achieving subsequent cascade targets, promoting initial test uptake remains the necessary first step.

## Conclusion

HIV self-testing uptake among MSM in Chongqing remains limited in the context of evolving testing policies. Uptake was associated with education level, marital status, sexual behavior, and stimulant use. Targeted, context-sensitive strategies, particularly those supporting men in stable partnerships and individuals with lower educational attainment may help expand early diagnosis and contribute to progress toward the UNAIDS testing targets.

## Data Availability

The raw data supporting the conclusions of this article will be made available by the authors, without undue reservation.
